# High Yield Transfer of Clean Large-Area Epitaxial Oxide Thin Films

**DOI:** 10.1007/s40820-020-00573-4

**Published:** 2021-01-04

**Authors:** Bowen Zhang, Chao Yun, Judith L. MacManus-Driscoll

**Affiliations:** Department of Materials Science and Metallurgy, University of Cambridge, 27 Charles Babbage Road, Cambridge, CB3 0FS UK

**Keywords:** Free-standing oxide thin films, High yield transfer, Wet etching, Crack prevention

## Abstract

In this work, we have developed a new method for manipulating and transferring up to 5 mm × 10 mm epitaxial oxide thin films. The method involves fixing a PET frame onto a PMMA attachment film, enabling transfer of epitaxial films lifted-off by wet chemical etching of a Sr_3_Al_2_O_6_ sacrificial layer. The crystallinity, surface morphology, continuity, and purity of the films are all preserved in the transfer process. We demonstrate the applicability of our method for three different film compositions and structures of thickness ~ 100 nm. Furthermore, we show that by using epitaxial nanocomposite films, lift-off yield is improved by ~ 50% compared to plain epitaxial films and we ascribe this effect to the higher fracture toughness of the composites. This work shows important steps towards large-scale perovskite thin-film-based electronic device applications.

**Supplementary information:**

The online version of this article (10.1007/s40820-020-00573-4).

## Highlights

- A new way to achieve high yield and large area oxide thin film transfer is developed. Three different film compositions are demonstrated: SrRuO_3_, CeO_2_, and CeO_2_/STO nanocomposite films.
- Cracks, wrinkles, and damages are prevented by the new transfer method. They are commonly introduced by conventional transfer processes.
- Vertically aligned nanocomposite (VAN) structures can further improve the transfer yield. Possible mechanisms related to increased fracture toughness are proposed.
- We have opened up a route to large-scale oxide thin-film-based electronic device applications.

**Supplementary information:**

The online version of this article (10.1007/s40820-020-00573-4).

## Graphical abstract

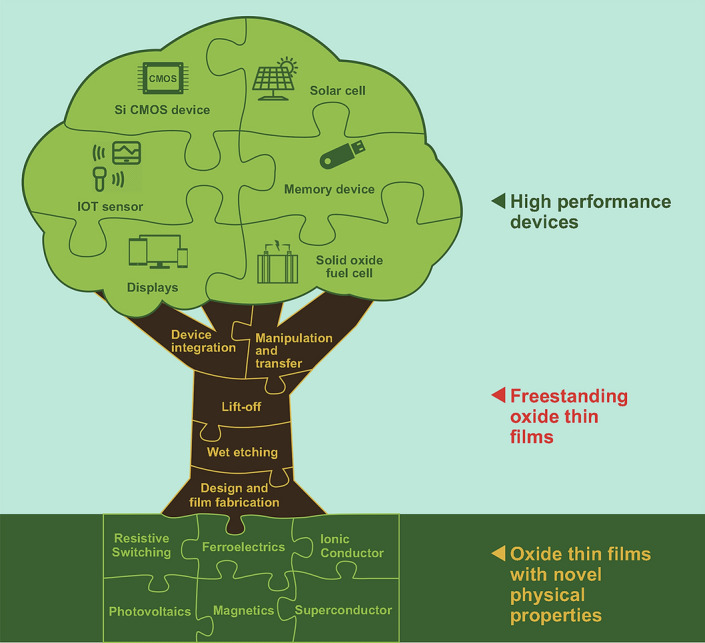

## Introduction

Functional oxide thin films are of great interest for their broad spectrum of physical properties, e.g. in dielectrics and ferroelectrics [1, 2], magnetics [3, 4], superconductors [5, 6], ionic conductors [7, 8], photovoltaics [9, 10], resistive switching [11, 12], etc. Furthermore, with the rapid development of two-dimensional (2D) materials and van der Waals heterostructures in the last decades, it is highly anticipated that exemplary functionalities will be achieved from low-dimensional, single-crystalline functional oxide thin films, particularly on flexible and semiconductor substrates [13]. In this way, it would be possible to integrate the unrivalled properties of low dimensional oxides with CMOS, and also obtain flexible sensors for IoT [14] and biological devices [15], and low-power consumer electronics [16], etc.

Single-crystalline oxide thin films are usually strictly bound to a limited range of hard substrates, which provide the appropriate lattice and thermal matching conditions for their epitaxial growth. Such substrates are usually inorganic crystals and therefore unable to bring mechanical flexibility to the films. The strong chemical bonds in the interface make it a challenge to separate the oxide thin films from the substrates, which limits their further applications.

Many approaches are reported to grow or transfer perovskite oxide thin films to a device-compatible substrate, including mechanical exfoliation [17, 18], van der Waals epitaxy [19–22], dry etching release methods [23–26], and wet etching release methods [27–32]. Among those methods, the wet etching release method has higher selectivity and is more cost-effective. It involves a selective chemical etching process to remove the substrate or sacrificial layers, and retains the high-quality growth of the epitaxial films. The sacrificial method typically results in less damage after transfer than the aforementioned methods [33]. Recently, there are many notable examples of free-standing perovskite thin films and superlattices prepared by dissolution of a sacrificial layer (Sr_3_Al_2_O_6_) in water inspired by the pioneering work by Hwang’s group [32, 34]. The resultant lifted-off films have minimized contaminant caused by the etchant solution. Hence, the wet etching method is arguably the most promising approach for producing free-standing single-crystalline oxide thin films for large-scale device applications.

In a typical wet etching release method, the first step is the detachment of the thin film from the substrate or sacrificial layer, which is then followed by the transfer process. While most current works are focusing on different etching methods, the upper limit of the film area and quality is usually dependent on the latter lift-off and transfer step. The key issue for a good transfer is to prevent the crack formation and minimize contamination. We note that while free-standing thin films of metals and some oxides have been shown to be relatively flexible [33, 35] compared to their bulk counterparts, it is clear that ceramic films are still brittle in thin film form and if crack initiation sites are present, the films will behave in a brittle manner [36, 37].

Existing thin-film transfer methods use well-developed transfer methods designed for graphene transfer. A support layer, e.g. PMMA [38], PDMS [39] or polystyrene (PS) [30], is employed and coated on the whole film surface before the wet etching process to prevent fracture. For oxide films, an important challenge during the transfer process is the introduction of cracks and tears. Also, in a typical graphene transfer process, the PMMA/graphene stack usually floats on the solution surface. This method cannot be simply applied to oxide thin films owing to their larger density and thickness, which makes it difficult to for surface floatation.

Several groups have explored the possibilities of the wet etching approach. In 2016, Bakaul et al. developed a PMMA-based method to transfer single-crystalline ferroelectric thin film onto Si-wafers at hundreds of micrometre length scale [40]. More recently, Shen et al. transferred a large area (5 × 10 mm^2^) thin film onto polyimide (PI) substrates by directly adhering the PI tape on thin film [41]. Ji et al. synthesized and transferred free-standing SrTiO_3_ and BiFeO_3_ ultrathin films down to one unit-cell via above-mentioned Sr_3_Al_2_O_6_ wet etching approach [39].

In this paper, a new way to achieve high yield, large-area (5 × 10 mm^2^, the maximum substrate area explored) oxide thin film transfer is developed. We use a wet chemical lift-off process using Sr_3_Al_2_O_6_ sacrificial layers, and a new PMMA-mediated transfer approach which involve attachment of a PET window layer to the PMMA, to provide extra rigidity and give easier handling of the film. The method avoids the physical damage introduced by the mechanical lifting process and gives a high yield transfer rate (~ 72%) onto Si- and flexible polymer (PET) substrates. Furthermore, we show that when epitaxial vertically aligned nanocomposite (VAN) films are used, the yield is further improved. Possible mechanisms related to an increased fracture toughness are proposed. Overall, three different film compositions/forms are demonstrated: SrRuO_3,_ CeO_2_, and CeO_2_/STO VAN. The successfully transferred films do not show macroscopic cracks. The lack of microscopic cracks is proven by showing minimal changes in resistivity of metallic SrRuO_3_ after lift-off compared to before.

## Experimental

### Film Fabrication by Pulsed Laser Deposition (PLD)

In this experiment, thin films are grown by pulsed laser deposition with a KrF excimer laser (*λ* = 248 nm). All the targets used in PLD are polycrystalline and prepared through solid-state reaction. For Sr_3_Al_2_O_6_ target, a stoichiometric mixture of SrO and Al_2_O_3_ powders was mixed together and then sintered in air at 1350 °C for 48 h with an intermediate grinding and pelletizing steps. For the CeO_2_ and SrRuO_3_ target, the raw powders were weighed to achieve a stoichiometric amount of mixture and were subsequently mixed and sintered using the same steps as we did for Sr_3_Al_2_O_6_ target. For the CeO_2_/STO target, CeO_2_ and SrTiO_3_ were mixed at a 50:50 molar ratio.

The substrate we used in this work is single-side polished SrTiO_3_ (001). Before deposition, the substrates were cleaned using an ultrasonic bath with different solutions, i.e. DI-H_2_O, acetone, and isopropanol for 10 min each. After that, the substrates were pre-annealed at an oxygen partial pressure (*p*_O2_) of 1 × 10^–5^ mbar for 30 min at 950 °C to achieve atomically flat single-terminated surfaces.

Then, a Sr_3_Al_2_O_6_ buffer layer was grown on the annealed SrTiO_3_ (001) substrate at a substrate temperature *T*_g_ = 700 °C and *p*_O2_ = 1 × 10^–6^ mbar, while using 1.25 J cm^−2^ laser fluence and a repetition rate of 1 Hz.

Finally, the target films, SRO, CeO_2_, and CeO_2_/STO nanocomposite films were grown in situ at *T*_g_ = 750 °C and *p*_O2_ = 0.2 mbar, using 1.5 J cm^−2^ laser fluence and a repetition rate of 2–5 Hz. After deposition, the films were post-annealed at 650 °C for 1 h under a *p*_O2_ of 0.4 bar to ensure equilibrium oxygen stoichiometry and to minimize the creation of oxygen vacancies inside the films.

### Exfoliation and Transfer of Thin Films

**Fig. 1:**
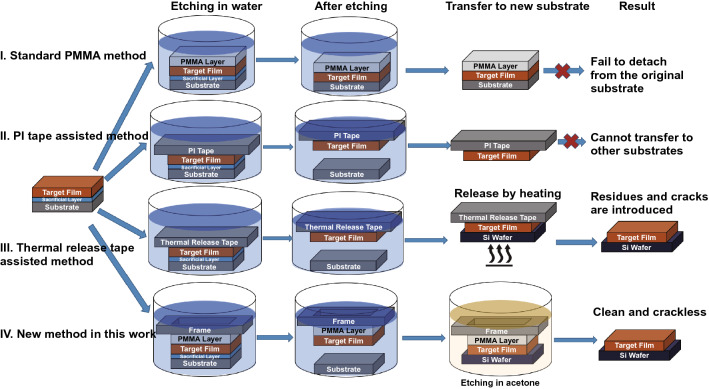
Schematics of four different wet-etching-based methods to fabricate oxide thin films. Methods I-III are known methods, and method IV is the new method developed in this work

In our new method VI, to load the support layer, a PMMA solution (Mw = 950 K, 4 wt% in anisole) was spin coated (2,000 RPM, 30 s) onto the thin film with the substrate and then naturally dried for 12 h to obtain a thin-film embedding structures with thickness about 300 nm. Then, a PET membrane was tailored as shown in Fig. 1 and then attached to the film (PMMA side) with moderate pressure. The thin film together with the PMMA layer and tape frame layer was immersed into room-temperature DI water to dissolve the Sr_3_Al_2_O_6_ buffer layer and remove the substrate. Before transfer, the Si wafer was processed by an oxygen plasma via reactive ion etching (RIE) in order to form a hydrophilic SiO_2_ layer and increase the adhesion of free-standing films. After etching in water, the supports with the thin films were placed on another substrate (e.g. Si wafer) and then soaked in acetone to dissolve the PMMA layer. The floating tape frame was then collected and disposed, while the thin film remained on the new substrate.

For the control group, we followed the method reported in Di Lu et al. [32] and Dianxiang Ji et al. [39] This is method III, the thermal release tape method, the most standard method for oxide film transfer. To transfer the free-standing oxide film to Si, the sample was stuck onto silicone-coated PET and released in the same manner (etching in water). After dissolving in water, the film/silicone-coated PET was attached to the new substrate. Finally, the free-standing film remained on the new substrate after peeling off the silicone-coated PET by heating at 70 °C for 10 min.

### Characterizations

AFM images were acquired in tapping mode using a Bruker Digital Instrument Nanoscope III. The SEM images and EDX analysis were done using an FEI Nova NanoSEM. The XRD data were taken using a high-resolution Bruker D8 with graded mirror and CuKα radiation. Data were recorded via 2*θ*–*ω* scans, with 2*θ* from 10° to 110°, step size = 0.01°, and the time for one scan is 1.5 h.

Electrical measurements are taken using a four-point probe station with a Keithley 2440 source-meter, with voltage applied to Pt electrodes of 0.1 mm diameter, formed by sputtering on the film surface. The thin-film samples were mounted with an in-line four-point probe configuration as shown in Fig. S3.

## Results and Discussion

### New Method to Transfer Epitaxial Oxide Thin Films

Figure 1 shows the four different methods based on the wet etching approach which have been studied for transferring single crystalline oxide thin films to Si wafers. The main difference between these methods is the support layer, which significantly influences the quality of final transferred films. Method I is the most commonly used 2D material transfer method, and it relies on the use of a sacrificial polymethyl-methacrylate (PMMA) film to support the 2D layers and to prevent them from folding or cracking during the etching and transferring process [42]. However, the PMMA approach is not applicable for oxide thin films as the films have high densities compared to 2D materials, which makes it difficult for the films to float on the surface and detach from the substrate. Besides, even 2D films of higher thickness have been reported to more likely to get broken during PMMA removal step [43]. In method II, PI tape (pressure sensitive adhesive tape) is used to support and protect the film. Although previous reported results showed that large area oxide thin film can be transferred to the PI tape, it is almost impossible to release the films to other non-adhesive substrates like Si wafers [41]. In method III, the sample is adhered to the thermal release tape or silicone-coated PET instead of the pressure sensitive tape in order to release the films by heating [32, 39]. However, it is difficult to obtain continuous coverage using this method. The appearance of voids, cracks, and some residues from the thermal release tape is inevitable after the transfer [44].

The last method, method IV, is a two-layer structure support method, and is newly introduced in this work. It combines the advantages of the PMMA-mediated transfer method [45] (i.e. prevention of film folding or cracking during transfer and gives almost continuous coverage) and the thermal release tape method (easy for manipulating large area films) [32]. As we already mentioned, oxide thin films are very brittle and fragile. Also, as already mentioned, oxide thin films are much denser than conventional 2D materials (e.g. CeO_2_ has a density of 7.22 g cm^−3^ as compared to graphene’s density of 2.27 g cm^−3^), and so they will not float on water.

The potential advantages of our new PMMA-mediated method in IV over other three standard methods of Fig. 1 are:

1. Reduction of film cracking. This is because the PET membrane is much more mechanically stable than the PMMA layer, allowing the free-standing film to be carefully manipulated, without flexing.
2. Film continuity. The spin-coated PMMA layer provides full coverage of the film and allowing the lifted-off film to be continuous. Besides, the spin-coated PMMA layer can be easily removed by acetone/chloroform.
3. Film flotation. The additional PET frame on the PMMA should stop the film from sinking. This is because the overall support + film density is < 1 g cm^−3^.
4. Reduction of film buckling. The rigidity of the frame structure should also reduce the effect of turbulence during the dissolution of PMMA.

The first step in the process of getting a perfect free-standing film is to grow the target single-crystal oxide thin film with a well-defined orientation. Several studies using pulsed laser deposition (PLD) and molecular-beam epitaxy (MBE) have demonstrated growth of single crystalline films on Sr_3_Al_2_O_6_ sacrificial layers [32, 39, 46, 47]. The layer is coherently strained to the STO when it is very thin (around 10 nm). Since Sr_3_Al_2_O_6_ is soluble in water, this avoids the use of acid etchants. Hence, it is the most suitable layer for most oxide thin films with perovskite structures [32]. In this work, we use PLD to grow the films on Sr_3_Al_2_O_6_ on (001) SrTiO_3_ single crystal substrates.

**Fig. 2:**
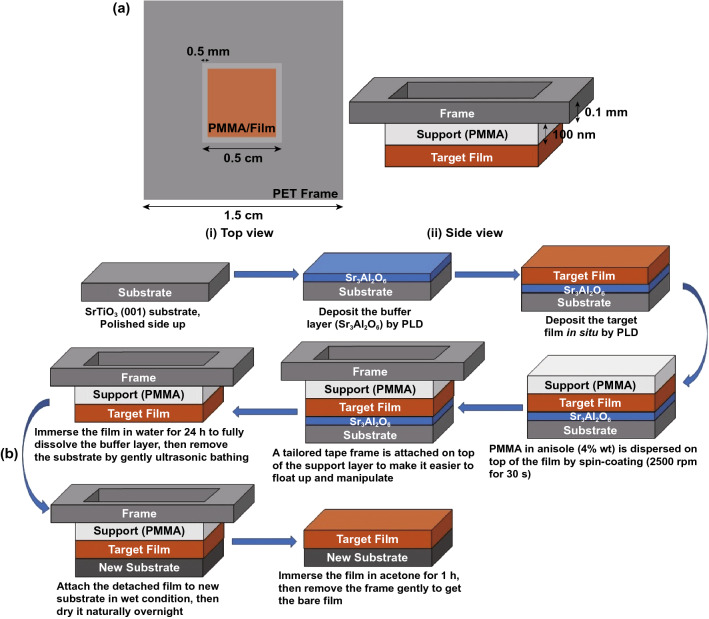
Schematic of PET frame on PMMA support for lifting off and placing a film on a new substrate. **a** Placement of PET frame on PMMA support. (i) Top view. (ii) Side view; **b** schematic of the whole process developed in this work based on method IV in Fig. 1

A sketch of the film with PMMA and PET membrane frame on top can be seen in Fig. 2a. After spin-coating a PMMA layer onto the film surface, a frame-shaped PET membrane is then attached on the top of the PMMA layer. (The experimental details are presented in Experimental Section.)

The whole thin film transfer process flow is shown in Fig. 2b. Three main parts are combined: (i) the epitaxial growth of Sr_3_Al_2_O_6_ sacrificial layer followed by in situ growth of target oxide thin film; (ii) lift-off process; (iii) transfer of film onto new substrate.

We successfully fabricated and transferred three different composition/structure oxide films to single crystal Si wafers: SrRuO_3,_ CeO_2_, and CeO_2_/STO nanocomposite films. In order to make sure all films are grown epitaxially with high quality, the films are grown on a 10-nm-thick Sr_3_Al_2_O_6_ buffer layer which preserves the perovskite step-and-terrace structure of underlying SrTiO_3_ substrate and this can be seen in AFM images (Fig. S1a, b).

We explored SrRuO_3_ because it has a perovskite structure and is metallic. The pseudocubic lattice parameter for SrRuO_3_ is 0.3923 nm which is quite similar to that of STO (0.3905 nm) with only − 0.46% lattice mismatch. Hence, it should grow coherently to the buffer with low interfacial defects. Since it is metallic, then after lift-off its structural integrity and connectivity can be assessed by undertaking electrical resistivity measurements.

We explored CeO_2_ as it is structurally mismatched (fluorite structure) to the buffer and will grow by domain matching epitaxy (DME) with a high concentration of misfit dislocations near the interface. This higher defect concentration may reduce structural integrity after lift-off, and thus, this system can be used to verify how effective the new transfer process is. To date, as far as known, only perovskite structured films have been grown and transferred by the Sr_3_Al_2_O_6_ buffer layer approach and so the process would be further validated by transfer of a non-perovskite film.

The CeO_2_/STO VAN films were tested to determine whether the presence of a nanostructured second phase within a film could help block crack growth (as composite structures are known to do so in the field of mechanical ceramics) [48–50] and thus to determine whether it is possible to enhance the yield of lifted-off films using composite systems. CeO_2_/STO VAN films were chosen as a good reference to the plain CeO_2_ films, and also because they have interesting ionic properties [8, 11, 51]. In these films, vertical nanocolumns of CeO_2_ grow embedded in a supporting matrix of SrTiO_3_ [51]. It is noted, however, that while composites could be advantageous on the one hand, and on the other hand, the Tb/inch^2^ density of vertical interfaces in VAN films may be defective, and so could lead to sites of crack initiation and degrade the mechanical properties.

### CeO_2_ Free-standing Films

For the CeO_2_ film, we compare lift-off using our new support method (no. IV in Figs. 1 and 2) with the thermal release tape methods (no. III in Fig. 1). We use no. III as the control method because it is most widely used for oxide thin film transfer [39, 47]. The other methods (I and II) are not suitable (and also not reported) for the transfer of oxide thin films to Si substrates.

**Fig. 3:**
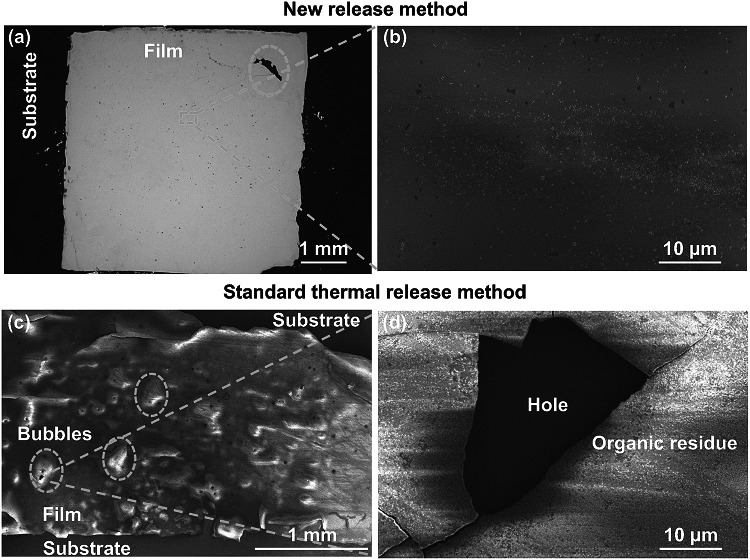
**a** Image of the surface of a CeO_2_ film (5 × 5 mm^2^) transferred by the new optimized method IV of Figs. 1 and 2. **b** SEM image of the CeO_2_ film. **c** Image of the surface of the film transferred by the thermal release tape assisted method III of Fig. 1. Bubbles due to heating process and cracks can be seen. **d** SEM image of the film. A thin layer of residual adhesives (white area) and a large hole can be seen. (Color figure onine)

We consider results for the CeO_2_ film first. In Fig. 3a, b, we show a 5 × 5 mm^2^ CeO_2_ single crystal thin film (100 nm thick) transferred to Si using our new method. The film is free from macroscopic cracks over an area of 4 × 4 mm^2^ with a small crack at top right corner (as seen from the SEM image in Fig. 3a). A very small amount of PMMA residue of < 100 nm is observed on the film surface (Fig. 3b). No organic polymer residual particles or layers were observed after searching the entire film area.

We compare the transferred film by our new method (Fig. 3a, b) to a film transferred by the thermal release tape transfer process. The SEM results are shown in Fig. 3c, d). We see that bubbles are introduced by the heating step, and large area organic adhesive residues are introduced by the adhesive. The mechanical forces from the peeling step also result in large cracks. These features are quite standard for the thermal release method [52].

### SrRuO_3_ Free-standing Films

**Fig.4:**
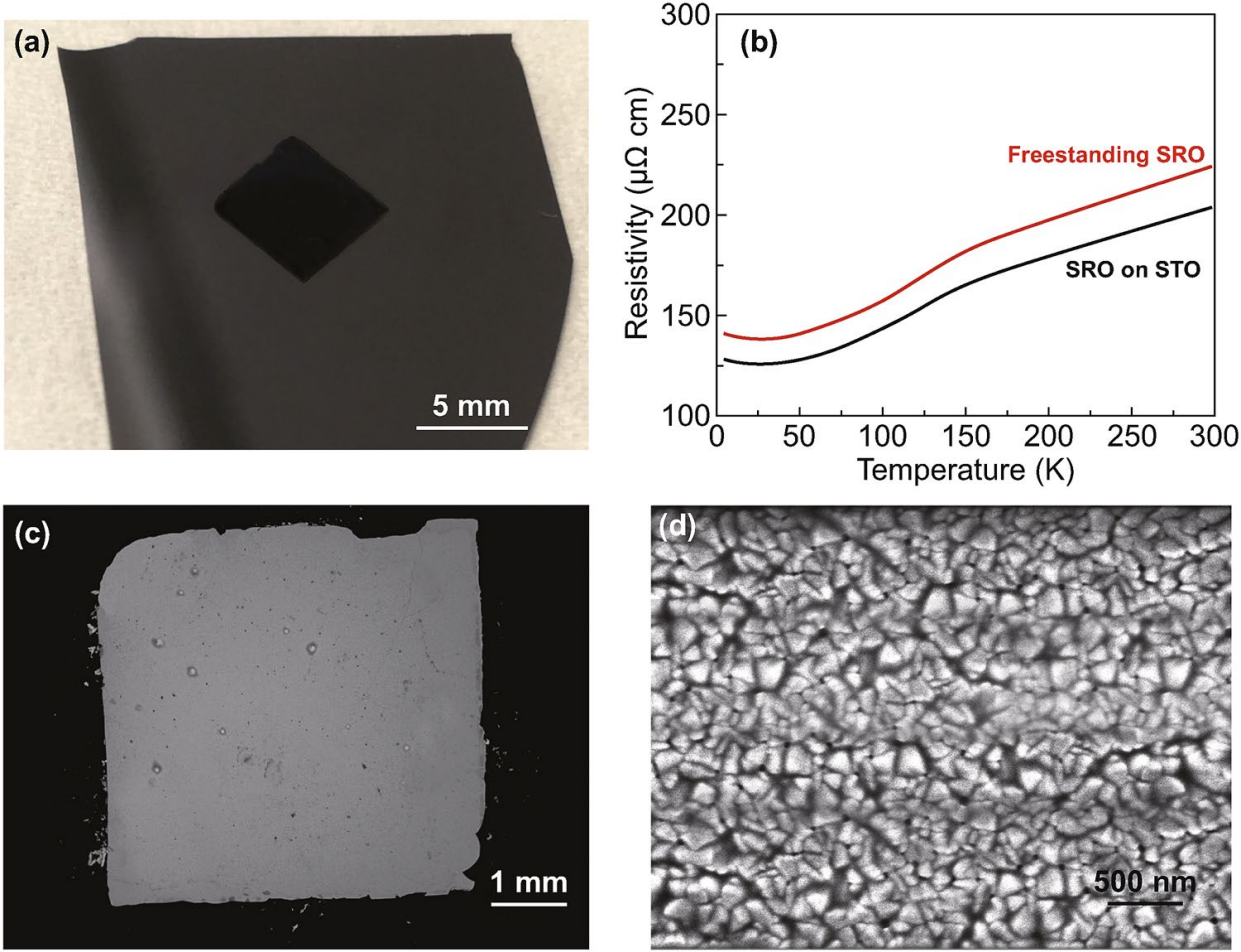
**a** Optical image of the surface of a SrRuO_3_ film (5 × 5 mm^2^) transferred by the optimized method IV of Figs. 1 and 2. **b** Resistivity measurements for the SrRuO_3_ film before and after transfer. **c, d** SEM image of the SrRuO_3_ film showing the surfaces of the columnar grains at the film surface

Next, we studied a lifted-off film of 100-nm-thick metallic SrRuO_3_ transferred to Si. We recall that SrRuO_3_ is well-lattice matched and fully structurally matched to the Sr_3_AlO_6_ films on SrTiO_3_. We explored the structural integrity of the film by measuring the electrical transport. Hence, if there are very tiny and/or buried cracks which are not easily observable with surface microscopy images, they would still lead to higher resistivity in the films. Figure 4 shows an image of a transferred film on Si (Fig. 4a) and the resistivity–temperature curve measured using a four-point probe method for the SRO thin film before and after transfer (Fig. 4b). Figure 4c, d shows magnified images of the film, revealing no macroscopic cracking. The resistivity (*ρ*) is only slightly increased from 203 to 223 μΩ cm (9.9%) at 297 K. This may be explained by the release of − 0.46% in-plane compressive strain in the film, as SrRuO_3_ is reported to have lower resistivity when compressively strained [53, 54]. However, small random cracks might also contribute to the marginally higher resistivity. Overall, the film performance is not degraded to any great extent after transfer, indicative of large-scale continuity and no influence of surface contamination from the transfer process. This contrasts with other reports which show larger increases in resistivity (e.g. 80% in LSMO reported by Di Lu et al. [32], ~ 700% in LSMO reported by Zengxing Lu et al. [46]).

### CeO_2_/STO Nanocomposite Free-standing Films

**Fig. 5:**
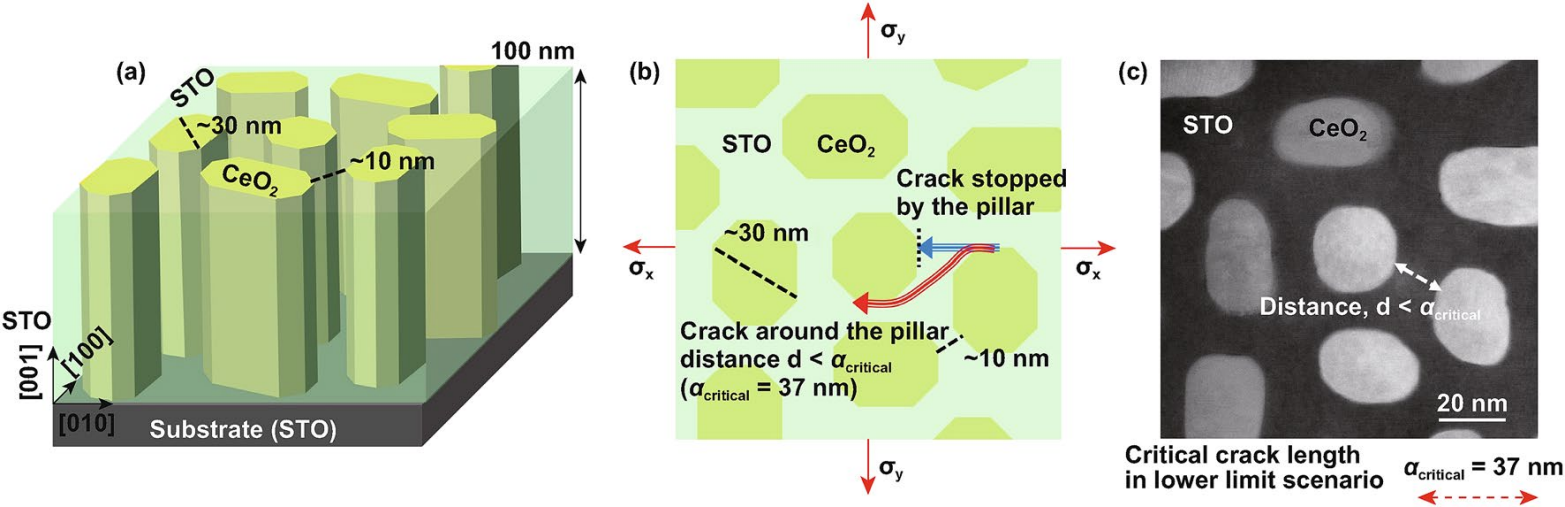
Schematic diagram of CeO_2_/STO nanocomposite film. **a** Side view showing the structure of the film. **b** Top view showing how 2 different cracks might propagate through the STO matrix. In scenario 1, a crack (blue) is stopped at a pillar. In scenario 2, a crack (red) propagates around the pillars. **c** Real surface scanning transmission electron micrograph of a CeO_2_/STO nanocomposite film. Image adapted from Zhu et al. [70]. (Color figure onine)

We now turn to the CeO_2_/STO VAN nanocomposite films. With these films, we aim to further explore the cleanliness of our new transfer process, but more importantly to also determine whether the complex VAN microstructure (a 3D schematic of the structure is shown in Fig. 5a, a surface schematic in Fig. 5b, and a real image in Fig. 5c) assists or hinders the transfer process.

**Fig. 6:**
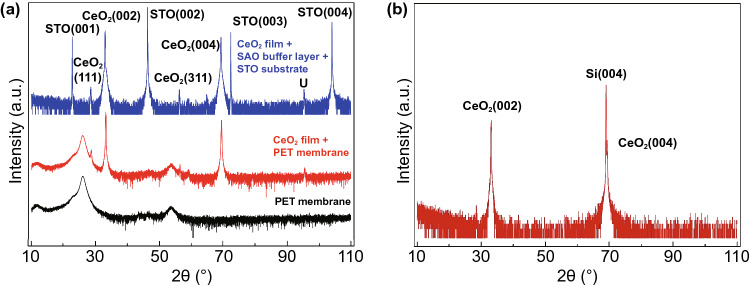
X-ray diffractograms (intensity on a log scale) for CeO_2_ film grown on Sr_3_Al_2_O_6_ buffer grown on SrTiO_3_ in different stages of transfer onto PET membrane or Si. **a** XRD 2*θ*–*ω* scan of CeO_2_ film before transfer, CeO_2_ film after transfer to PET, and blank PET membrane. *U* = unidentified peak. **b** XRD 2*θ*–*ω* scan of CeO_2_ film transferred to Si wafer

To be sure of the validity of any positive VAN results, we studied transfer onto both Si and flexible PET. We compare to the results to the plain CeO_2_ films. First, we investigated the influence of any chemical reaction effects. In Fig. 6a, we show X-ray diffraction (XRD) 2*θ*–*ω* scans of the CeO_2_ film before and after transferring to PET. The blank PET membrane is also shown. After transfer, all the STO substrate peaks and Sr_3_AlO_6_ buffer peaks have gone, while all CeO_2_ peaks have been preserved. This is as expected for successful selective dissolution and transfer. The CeO_2_ (002) and (004) peaks show that the aligned crystalline structure is retained after the transfer process. Minor CeO_2_ (111) and CeO_2_ (311) peaks are also present (~ 1/1000 intensity of main (002) peak), in both the untransferred and transferred film.

Figure 6b shows the XRD 2*θ*–*ω* scan of the CeO_2_ film transferred to Si. Similar to Fig. 6a, the CeO_2_ (002), CeO_2_ (004), and Si (004) are also clearly observed. The peak positions, shapes, and intensities are preserved for the transferred CeO_2_ membrane compared to the as-grown film. As shown in Fig. S2, the XRD pattern for the CeO_2_/STO VAN film shows the same effect as the CeO_2_ thin films, i.e. that all of the film peaks remain the same after lift-off. Hence, for the VAN film clear film CeO_2_ and STO matrix peaks are present both before and after lifting off the STO substrate onto the PET substrate.

**Fig. 7:**
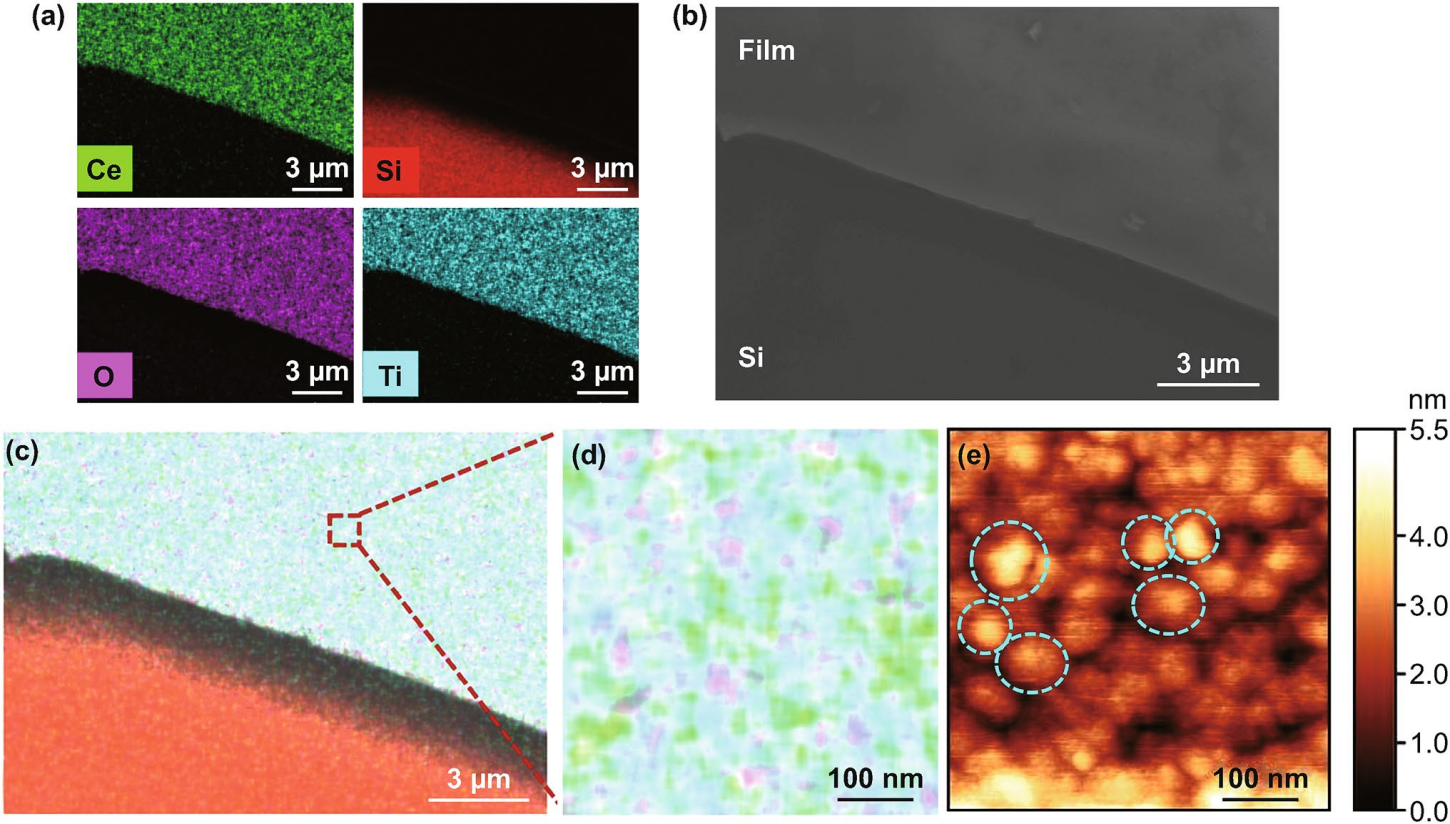
Top view SEM and AFM images of transferred VAN CeO_2_/STO film on a Si substrate. **a** EDS images. **b** SEM image. **c**, **d** show blow ups of EDS image for all-element mapping showing clear nanopillars (light green dots) of CeO_2_ embedded in STO matrix of film after transfer. **e** AFM image of the surface morphology of the film. Some nanopillars are circled with green dashes. The nanopillar structure of CeO_2_ is observed in both the EDS image of (**d**) and the AFM image of (**e**) and is perfectly preserved after transfer. (Color figure onine)

Top view SEM image and EDS elemental distribution maps of the CeO_2_/STO VAN film transferred onto Si are shown in Fig. 7. The bottom left region of the elemental maps in Fig. 7a shows the Si substrate, i.e. there is a high intensity of Si. A clear boundary is observed between the Si and the VAN CeO_2_/STO film at the upper right regions of the maps, where Ce, Ti, and O are observed in high intensity. Figure 7b shows an SEM image of the film on the Si, showing the film uniformity and again, clear boundary. An all element map is shown in Fig. 7c, with an enlarged area in Fig. 7d. Circular regions of high concentration of Ce are observed surrounded by regions with no Ce. This confirms the formation of the CeO_2_ pillars (circles in 2D) in the SrTiO_3_ matrix. An AFM image showing the same pillar features is shown in Fig. 7e. The diameter of the nanopillar (CeO_2_) is observed to be around ~ 30 nm in both Fig. 7d, e, in agreement with the TEM image of Fig. 1c. The columnar structure of film is not degraded by the transfer process and is very smooth (root mean square (RMS) roughness of 0.88 nm, obtained by squaring each height value in the dataset, then taking the square root of the mean).

### Higher Yield Transfer Obtained from Nanocomposite Structure

**Fig. 8:**
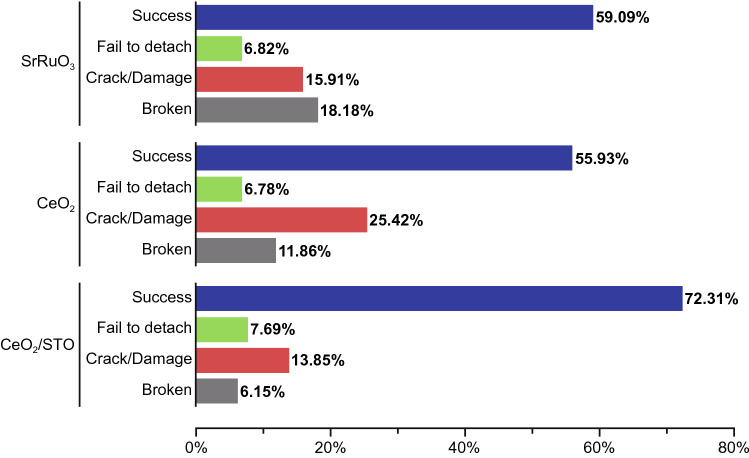
Distribution of different result types from each thin film composition/form

We now explore the efficacy of our new transfer method and also whether VAN films improve or degrade the process. In Table 1 and Fig. 8, we show the transfer success rates and other transfer characteristics. We divided the success information into 4 groups (successful transfers, and then, if unsuccessful, the nature of this, i.e. ‘Fail to detach’, ‘Crack/damage’ or ‘Broken’). ‘Fail to detach’ indicates the film did not get fully removed from the substrate. The last 2 groupings relate to formation of wrinkles, cracks, and folds introduced by the transfer process. Films had obvious cracks or damage are in the “Crack/Damage” group, whereas in the ‘Broken into Pieces’ group, the films were cracked into small pieces.

**Table 1:** Statistics of different result types from each thin film composition

SrRuO_3_		CeO_2_		CeO_2_/STO	
Number	Percentage	Number	Percentage	Number	Percentage
26	59.09%	33	55.93%	47	72.31%
3	6.82%	4	6.78%	5	7.69%
7	15.91%	15	25.42%	9	13.85%
8	18.18%	7	11.86%	4	6.15%
44	100%	59	100%	65	100%

The overall yields for SrRuO_3_, CeO_2_, and CeO_2_/STO are 59.09%, 55.93%, and 72.31%, respectively. It is clear that the CeO_2_/STO VAN nanocomposite films have a higher yield (72.31%) in the transfer process with less crack/damage (13.85%) or breakage (6.15%).

From a fracture toughness point of view, combining SrTiO_3_ with CeO_2_ should make the system more brittle owing to lower fracture toughness of SrTiO_3_ [55, 56]. However, our composite films are not more brittle as compared to the single-phase plain films: films can be transferred without cracks and the transfer yield is higher. Therefore, this improvement in the composite films must be due to (an) other mechanism(s). We consider key possible mechanisms below.

Assuming no handling stresses in the films after lift-off, the stress will originate from the residual stress induced by the lattice mismatch. CeO_2_ has a cubic fluorite structure with lattice constant *a* = 0.5411 nm. Epitaxial (001) CeO_2_ is expected to grow with its [100] direction in [110] direction of the STO (001) substrate, i.e. the in-plane crystalline orientations of CeO_2_ are rotated by *φ* = 45° with respect to STO. Therefore, CeO_2_ has an effective lattice parameter *a* = 0.5411/√2 ≈ 0.3826 nm, which resulting a 2% lattice mismatch with STO substrates (*a* = 0.3905 nm). The residual stress between plain CeO_2_ and the STO substrate can be up to 3.3 GPa, as reported by Aline Fluri et al. [57] This represents an upper limit stress because the STO matrix component in the film (~ 50% volume fraction) has no lattice mismatch with the substrate.

The key question is what is the critical crack size is for this level of in-plane biaxial stress. We assume an infinite plate with a microcrack under biaxial tensile stress (shown by the red arrows in Fig. 5b). We need to determine whether the presence of the CeO_2_ nanopillars will prevent the critical crack size in the VAN films from being reached. To estimate this, we turn to the Griffith crack criterion for brittle materials which indicates that when a crack reaches a certain critical length, the crack will propagate unstably. If it is prevented from reaching this value, it will be stable [58]:

1 $$K_{{{\text{IC}}}} = 1.12\sigma_{{{\text{critical}}}} \sqrt {\pi a_{0} }$$

2 $$a_{{{\text{critical}}}} = 2a_{0}$$

*K*_IC_ is the plane strain fracture toughness, *a*_0_ is the length of edge crack, *σ*_critical_ and *a*_critical_ are the critical stress and critical crack lengths. *a*_*0*_ represents the length of a microcrack in the film.

Here *K*_IC_ for CeO_2_ [55] and STO [56] is 1.3 and 0.89 MPa m^1/2^, respectively. From Eqs. (1) and (2), *a*_critical_ of CeO_2_ and STO is calculated to be 78 and 37 nm, respectively.

*a*_critical_ for the STO film matrix is 37 nm and this < the shortest distance, *d*, between the nanopillars in the matrix. Hence, the crack could be blocked from reaching *a*_critical_. But this will only occur if the energetics at the crack tip allow this. There are several possibilities for making the crack opening less favourable. (1) The CeO_2_ nanopillar regions has a higher *K*_IC_ (~ 50% higher for CeO_2_ than STO) which will increase the toughening. (2) There is a room-temperature phase transition in nano-CeO_2_ films [59] which could be activated by the stress at the crack tip. This phase transition could then absorb the energy at the tip and arresting its progression. This situation is similar to the phase transformation toughening shown by ZrO_2_ particles embedded in ceramics [60–62]. This crack blocking by nanopillar is schematically shown as Scenario 1 (blue crack) in Fig. 5b.

We also note, however, that there will be cracks of less favourable orientation than Scenario 1 which pass directly between two nanopillars. In Scenario 2 (Fig. 5b), a crack can weave through the STO matrix, by moving around the nanopillars. Here, several different toughening mechanisms can come into effect to increase the resistance to crack propagation, as in conventional composite ceramics, where finely dispersed nanoparticles in a matrix give significantly enhanced fracture toughness [49, 63–65]. These include particle–matrix interfacial debonding, microvoiding, matrix shear yielding, crack bridging, crack deflection and increased tortuosity [66–69].

Since the structural mismatched vertical interface is more defective than the CeO_2_ pillars themselves [70], the cracks in the STO will pass *around* the weaker CeO_2_ pillars rather than through them. When *a*_critical_ is reached, then catastrophic failure would occur. For *a*_critical_ of ~ 37 nm, as shown in Fig. 5b, the crack would pass around 2 nanopillars. The length for this circular crack can be estimated as 2 semi-circles, i.e. length *l* = (*π*/2) *d*, where d is the distance between start and end. The extra length for this crack (from start to end) compared to a straight crack is ~ 57%, and hence, the energy to fracture because of this tortuosity is higher by this amount also. Moreover, a tortuous crack path means that the stress will be less effective in opening up the crack tip as the stress will not be perpendicular to the crack in all regions.

Overall, by invoking different fracture mechanisms of either prevention of crack propagation, inducing a higher fracture energy associated with a more tortuous crack path, and/or reduction of the operative stress, the higher yield of the transfer process obtained for the VAN films compared to plain films can be understood.

## Conclusions

In summary, we have developed a new method, based on PMMA membranes, for transferring large-area (up to 5 × 10 mm^2^, so far) oxide thin films from SrTiO_3_ substrates onto different substrates. Three different types of thin films (in terms of crystal structure and film form—plain or composite) were successfully transferred with good yield and quality. Cracks, wrinkles, and damages which are commonly introduced by conventional transfer processes, are prevented by the new transfer method. Furthermore, by adding STO into CeO_2_ to form a nanocomposite structure, we showed improved lift-off yield rates by ~ 50%. Overall, we have demonstrated two approaches to significantly improve the transfer process of free-standing 2D single-crystalline functional oxide materials. The work has the potential to enable a wide range of oxide films to be transferred to different substrates for next-generation low-dimensional electronic devices.

## Supplementary information

Supplementary file 1 (PDF 312 kb)
