# Supplementary material for: High Yield Transfer of Clean Large-Area Epitaxial Oxide Thin Films
Source: Nanomicro Lett. 2021 Jan 4;13:39. doi: 10.1007/s40820-020-00573-4 (PMC8187697; doi:10.1007/s40820-020-00573-4)
Supplement: Supplementary file 1 — (PDF 312 kb) [file 40820_2020_573_MOESM1_ESM.pdf]

Supporting Information for

High Yield Transfer of Clean Large-Area Epitaxial Oxide Thin Films

Bowen Zhang[1], Chao Yun[1], Judith L MacManus-Driscoll[1, *]

[1]Department of Materials Science and Metallurgy, University of Cambridge, 27 Charles Babbage Road, Cambridge CB3 0FS, United Kingdom

*Corresponding author. E-mail: jld35@cam.ac.uk (Judith L MacManus-Driscoll)

Supplementary Figures

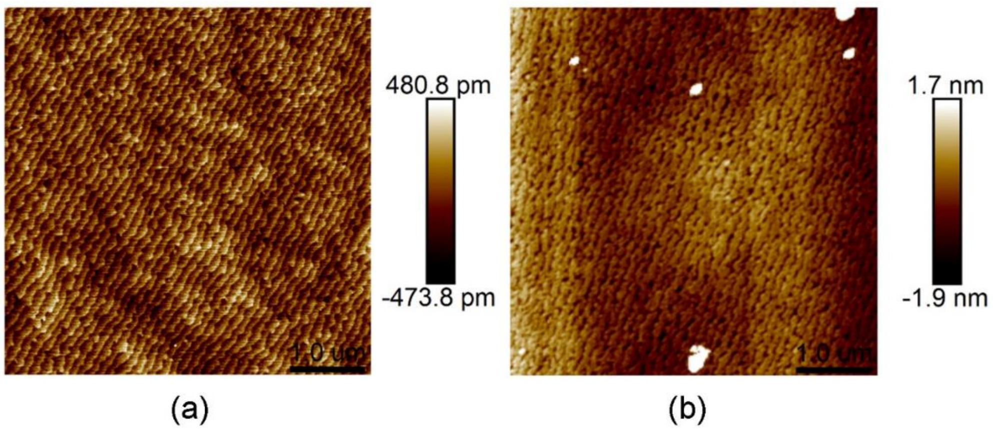

Fig. S1 AFM image of the surface morphology, showing the step-and-terrace structure of $SrTiO_3$ substrate (**a**) and 10 nm thick $Sr_3Al_2O_6$ film on the substrate (**b**)

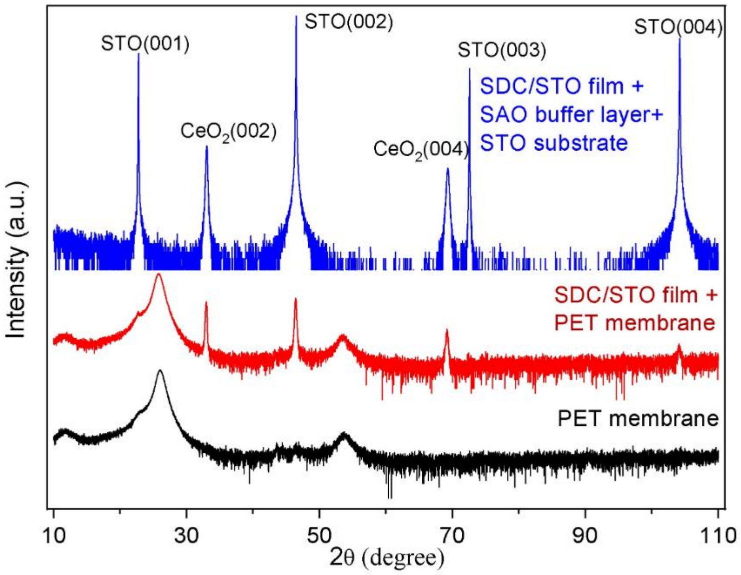

Fig. S2 XRD $2\theta - \omega$ pattern (log scale) of the CeO_2/STO VAN nanocomposite film before and after transfer. The remaining STO peaks after transfer proves the existence of STO matrix phase

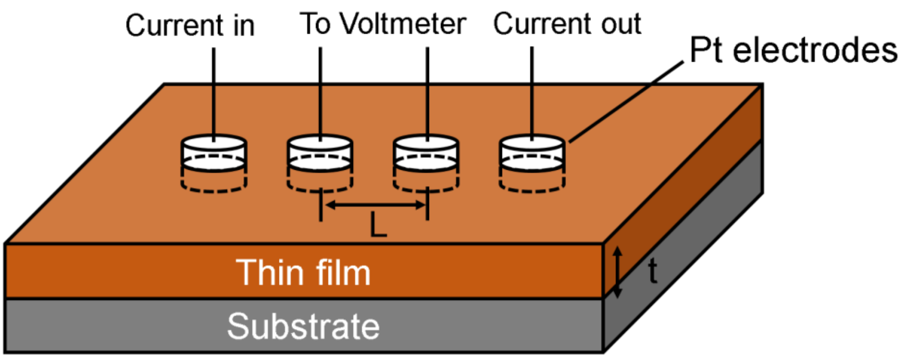

Fig. S3 Illustration of four-probe resistance measurements for measuring the resistivity of the $SrRuO_3$ film
